# STAT3 inhibition with galiellalactone effectively targets the prostate cancer stem-like cell population

**DOI:** 10.1038/s41598-020-70948-5

**Published:** 2020-08-18

**Authors:** Giacomo Canesin, Valentina Maggio, Macarena Palominos, Anna Stiehm, Hector R. Contreras, Enrique A. Castellón, Juan Morote, Rosanna Paciucci, Norman J. Maitland, Anders Bjartell, Rebecka Hellsten

**Affiliations:** 1 Division of Urological Cancers, Department of Translational Medicine, Skåne University Hospital Malmö, Lund University, Malmö, Sweden; 2 Department of Surgery, Cancer Research Institute, Beth Israel Deaconess Medical Center, Harvard Medical School, Boston, MA USA; 3 Biomedical Research and Translational Oncology Unit, Vall d’Hebron Research Institute, Barcelona, Spain; 4 Department of Basic and Clinic Oncology, Faculty of Medicine, University of Chile, Santiago, Chile; 5 Cancer Research Unit, Department of Biology, University of York, Heslington, York, North Yorkshire UK

**Keywords:** Cancer, Stem cells, Urology

## Abstract

Cancer stem cells (CSCs) are a small subpopulation of quiescent cells with the potential to differentiate into tumor cells. CSCs are involved in tumor initiation and progression and contribute to treatment failure through their intrinsic resistance to chemo- or radiotherapy, thus representing a substantial concern for cancer treatment. Prostate CSCs’ activity has been shown to be regulated by the transcription factor Signal Transducer and Activator of Transcription 3 (STAT3). Here we investigated the effect of galiellalactone (GL), a direct STAT3 inhibitor, on CSCs derived from prostate cancer patients, on docetaxel-resistant spheres with stem cell characteristics, on CSCs obtained from the DU145 cell line in vitro and on DU145 tumors in vivo. We found that GL significantly reduced the viability of docetaxel-resistant and patient-derived spheres. Moreover, CSCs isolated from DU145 cells were sensitive to low concentrations of GL, and the treatment with GL suppressed their viability and their ability to form colonies and spheres. STAT3 inhibition down regulated transcriptional targets of STAT3 in these cells, indicating STAT3 activity in CSCs. Our results indicate that GL can target the prostate stem cell niche in patient-derived cells, in docetaxel-resistant spheres and in an in vitro model. We conclude that GL represents a promising therapeutic approach for prostate cancer patients, as it reduces the viability of prostate cancer-therapy-resistant cells in both CSCs and non-CSC populations.

## Introduction

Prostate cancer (PCa) is one of the main leading causes of cancer-related mortality and the most frequently diagnosed cancer in men^1^. The conventional therapy for early stage PCa includes surgical removal of the prostate and hormonal ablation by androgen deprivation therapy (ADT), which is effective in many cases^2,3^. However, these therapies often fail due to an incomplete depletion of tumor cells, resulting in tumor relapse and in patients developing castration-resistant PCa (CRPC), for which there are currently few therapeutic options^4,5^.

There is increasing evidence that most human cancers are driven by a rare population of transformed cells with stem and progenitor properties, defined as cancer stem cells (CSCs)^6,7^. CSCs represent a small subpopulation of largely quiescent cells that reside within tumors and have the potential to differentiate into recognizable, replicating tumor cells; therefore they are considered major elements in tumor initiation, growth and progression, metastasis and tumor heterogeneity^8,9^. Accumulating evidence indicates that PCa CSCs are characterized by the expression of specific cell surface markers, including CD133 and CD44, and are less differentiated than transient-amplifying (TA) or committed-basal (CB) cells^10–12^. These CSCs are able to self-renew and show high clonogenic ability, which makes them key drivers in the oncogenic and metastatic process^13,14^. Due to their resistance to conventional therapies, CSCs are related to tumor recurrence after chemo- and radiotherapy: for this reason, targeting CSCs has been suggested as a potential novel therapy to suppress PCa metastasis^15,16^. Gene expression profiling of PCa CSCs from primary cultures revealed a significant role for signal transducer and activator of transcription 3 (STAT3) in regulating the stem cell niche, indicating that STAT3 inhibition could be a valid strategy to selectively target PCa-CSC^11,17–19^.

Our group has previously characterized galiellalactone (GL) as a direct STAT3 inhibitor that binds to STAT3 to prevent the transcription of its target genes without interfering with its upstream activation^20^. Blockade of STAT3 by GL not only reduces proliferation and induces apoptosis of PCa cells and ALDH1A1 expressing cells in vitro, it also inhibits the growth of prostate tumors and the metastatic spread to regional and distal lymph nodes in vivo^21,22^. In this study we investigated the effect of GL on PCa CSCs in several models, including patient-derived primary tumor cultures and docetaxel resistant PCa cell spheres, in order to better characterize this compound as a therapeutic approach for PCa patients.

## Results

### Characterization of stem cell and transient amplifying cell populations from DU145 cells

**Figure 1:**
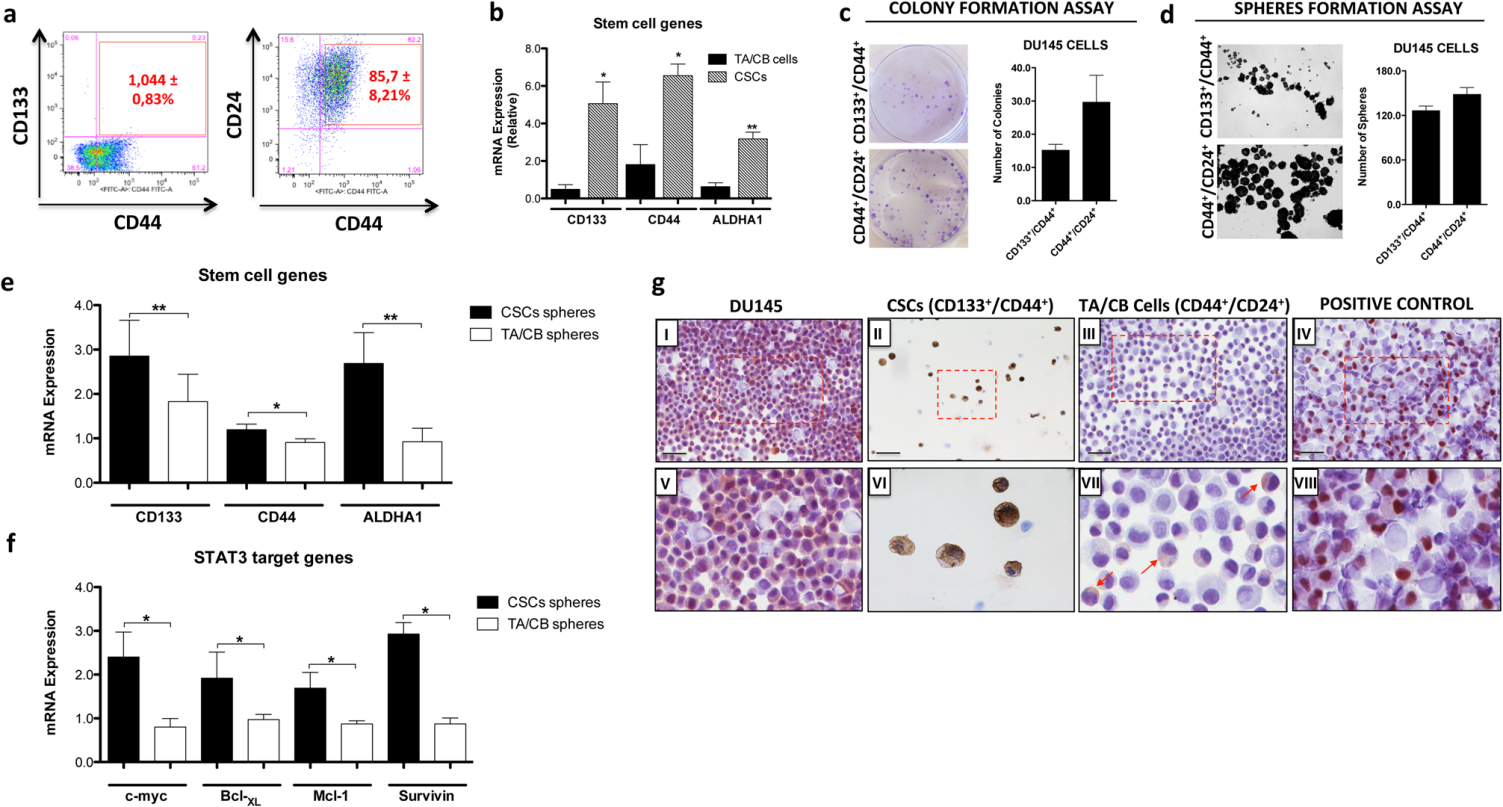
Characterization of stem cell and transient amplifying/committed basal cell populations from DU145 cells. (**a**) Flow cytometry analysis of CSCs (CD133^+^CD44^+^) and TA/CB (CD44^+^CD24^+^) populations in DU145 cells. (**b**) qPCR analysis of CD133, CD44 and ALDH1A1 gene expression in CSCs and TA/CB cells sorted from DU145 cells. Results represent the mean ± s.e.m. of five independent experiments (n = 5), each performed in triplicate. *p < 0.05; **p < 0.01. (**c**) Colony formation assay of CSCs and TA/CB cells sorted from DU145 cells and grown on collagen-coated plates. Representative images are shown on the left; the number of colonies is shown in the right graph. Results represent the mean ± s.e.m of three (n = 3) independent experiments, each performed in duplicate. (**d**) Sphere formation assay on CSCs and TA/CB cells sorted from DU145 cells. Representative images are shown on the left; the number of spheres is shown in the right graph. Results represent the mean ± s.e.m of three (n = 3) independent experiments, each performed in triplicate. (**e**) Gene expression analysis of stemness related genes in spheres derived from CSCs (CD133^+^/CD44^+^) and TA/CB (CD44^+^/CD24^+^) populations. Gene expression was studied by qPCR for CD133, CD44 and ALDH1A1. Results represent the mean ± s.e.m of three (n = 5) independent experiments, each performed in triplicate. *p < 0.05; **p < 0.01. (**f**) Gene expression analysis of STAT3-target genes in spheres derived from CSCs (CD133^+^/CD44^+^) and TA/CB (CD44^+^/CD24^+^) populations. Gene expression was studied by qPCR for c-myc, Bcl-_XL_, Mcl-1 and Survivin. Results represent the mean ± s.e.m of three (n = 3) independent experiments, each performed in triplicate. *p < 0.05. (**g**) Immunocytochemical analysis of phosphorylated STAT3 (pSTAT3) levels in DU145 cells (panel I), CSCs (panel II) and TA/CB cells (panel III) sorted from DU145 cells. LNCaP cells treated with 50 ng/ml IL-6 were used as positive control (panel IV). Panels V to VIII represents enlargements of the area in the red square. Arrows in (VII) indicate positive cytoplasmic staining. Scale bar = 100 μm.

We first determined the characteristics of stem and non-stem cell populations in DU145 cells, based on the expression of stem (CD133), basal (CD44) and luminal (CD24) cell surface markers. Flow cytometry analysis showed that CSCs (CD133^+^/CD44^+^) were detected as a small population in DU145 cells (1,044%), while the most abundant population (85.74%) was represented by TA/CB cells (CD44^+^/CD24^+^) (Fig. 1a). We then sorted CSCs and TA/CB populations based on the expression of the same surface markers and sought to determine their characteristics. Sorted CSCs showed higher expression levels of the stemness genes CD133, CD44 and ALDH1A1, compared to TA/CB cells (Fig. 1b). Furthermore, we found that cells from both populations were able to form colonies (Fig. 1c) and spheres (Fig. 1d) when grown in vitro. Notably, spheres derived from CSCs (CD133^+^/CD44^+^) showed higher expression of CD133, CD44 and ALDH1A1 (Fig. 1e) as well as higher levels of the STAT3-target genes c-myc, Bcl-_XL_, Mcl-1 and Survivin compared to spheres derived from TA/CB cells (CD44^+^/CD24^+^) (Fig. 1f). Furthermore, immunocytochemistry analysis on sorted cells from DU145 showed that CSCs express higher levels of active phosphorylated STAT3 (pSTAT3) compared to the TA/CB population (Fig. 1g). Altogether, these data indicate that CD133^+^/CD44^+^ cells and their derived spheres retain stemness characteristics and show high activity of STAT3, in agreement with previously published work^11,21^.

### Effect of GL on sorted CSC and TA/CB cell populations

**Figure 2:**
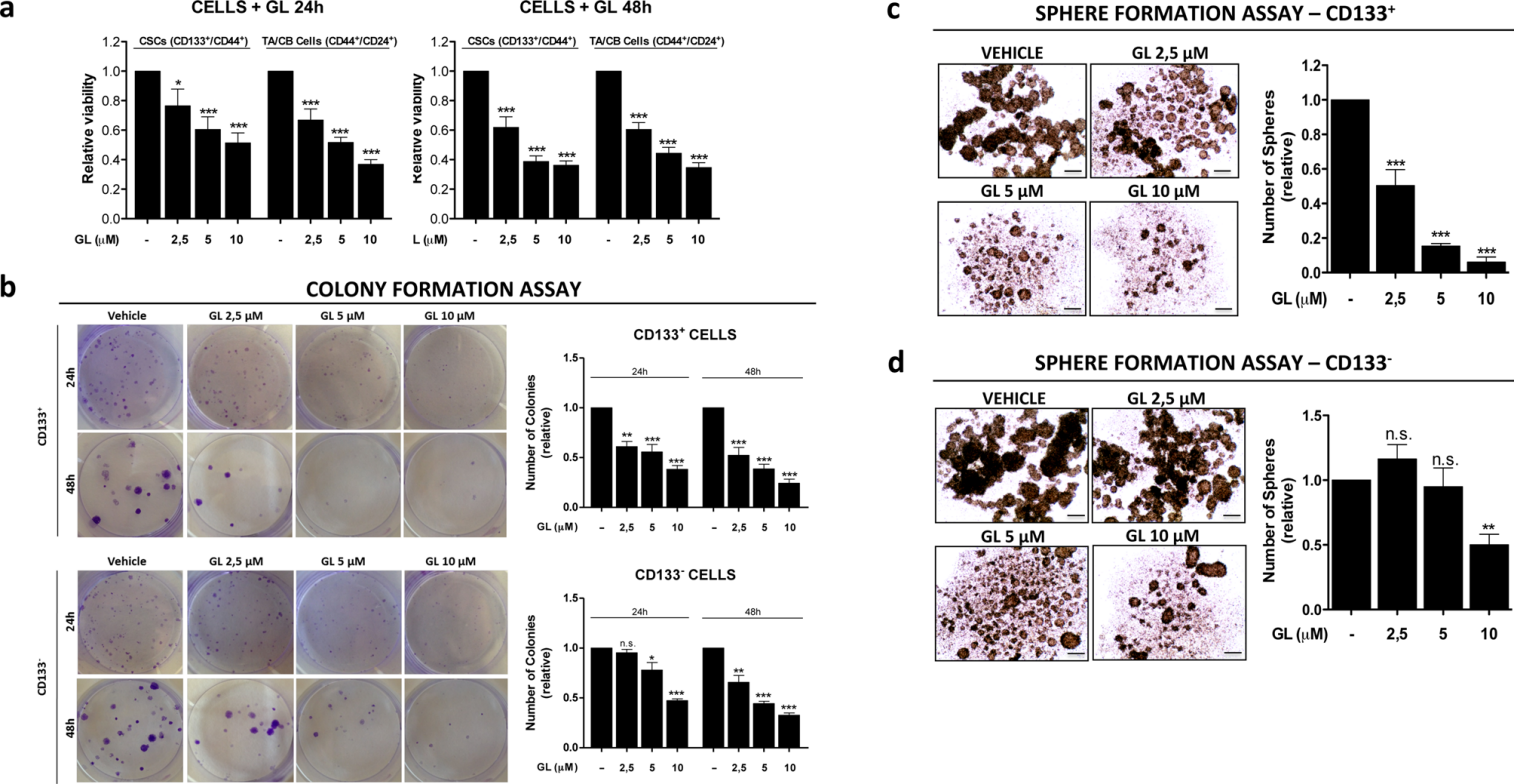
Effect of GL on the viability, colony and sphere formation ability of CSCs and TA/CB cells. (**a**) Viability assay on CSCs and TA/CB cells sorted from DU145 cells and treated with vehicle or 2.5–10 μM GL for 24 or 48 h. Results represent the mean ± s.e.m of six (n = 6) independent experiments, each performed in triplicate. Statistical significance was determined using one-way ANOVA with Bonferroni post hoc test. *p < 0.05; ***p < 0.001. (**b**) Colony formation assay of CSCs (CD133^+^, top) and TA/CB (CD133^−^, bottom) cells sorted from DU145 cells and grown on collagen-coated plates for 24 or 48 h in the presence of vehicle or 2.5–10 μM GL. Representative images are shown on the left; the number of colonies is shown in the right graph. Results represent the mean ± s.e.m of three (n = 3) independent experiments, each performed in triplicate. Statistical significance was determined using one-way ANOVA with Bonferroni post hoc test. *p < 0.05; **p < 0.01; ***p < 0.001; *n.s.* not significant. (**c**) Sphere formation assay on CSCs cells sorted from DU145 cells and grown in the presence of vehicle or 2.5–10 μM GL. Representative images are shown on the left; the number of CSCs-derived spheres is shown in the right graph. Results represent the mean ± s.e.m of three (n = 3) independent experiments, each performed in triplicate. Statistical significance was determined using one-way ANOVA with Bonferroni post hoc test. ***p < 0.001. (**d**) Sphere formation assay on TA/CB cells sorted from DU145 cells and grown in the presence of vehicle or 2.5–10 μM GL. Representative images are shown on the left; the number of CSCs-derived spheres is shown in the right graph. Results represent the mean ± s.e.m of three (n = 3) independent experiments, each performed in triplicate. Statistical significance was determined using one-way ANOVA with Bonferroni post hoc test. **p < 0.01; *n.s.* not significant.

**Figure 3:**
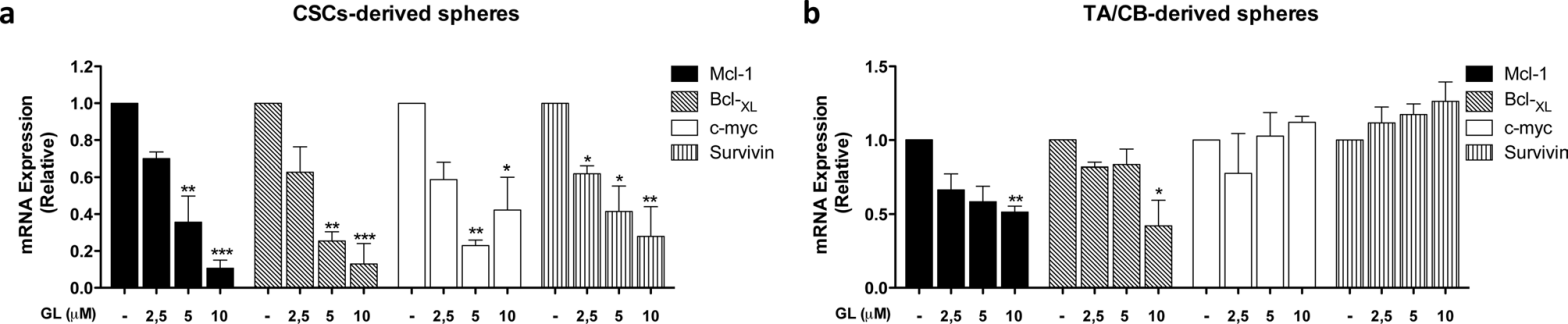
Effect of GL on the expression of STAT3-target genes. (**a**, **b**) qPCR analysis of Mcl-1, Bcl-_XL_, c-myc and survivin gene expression in CSCs-derived spheres (**a**) and in TA/CB-derived spheres (**b**) grown in the presence of vehicle or 2.5–10 μM GL. Results represent the mean ± s.e.m. of three independent experiments (n = 3), each of which was performed in triplicate. *p < 0.05; **p < 0.01; ***p < 0.001.

**Figure 4:**
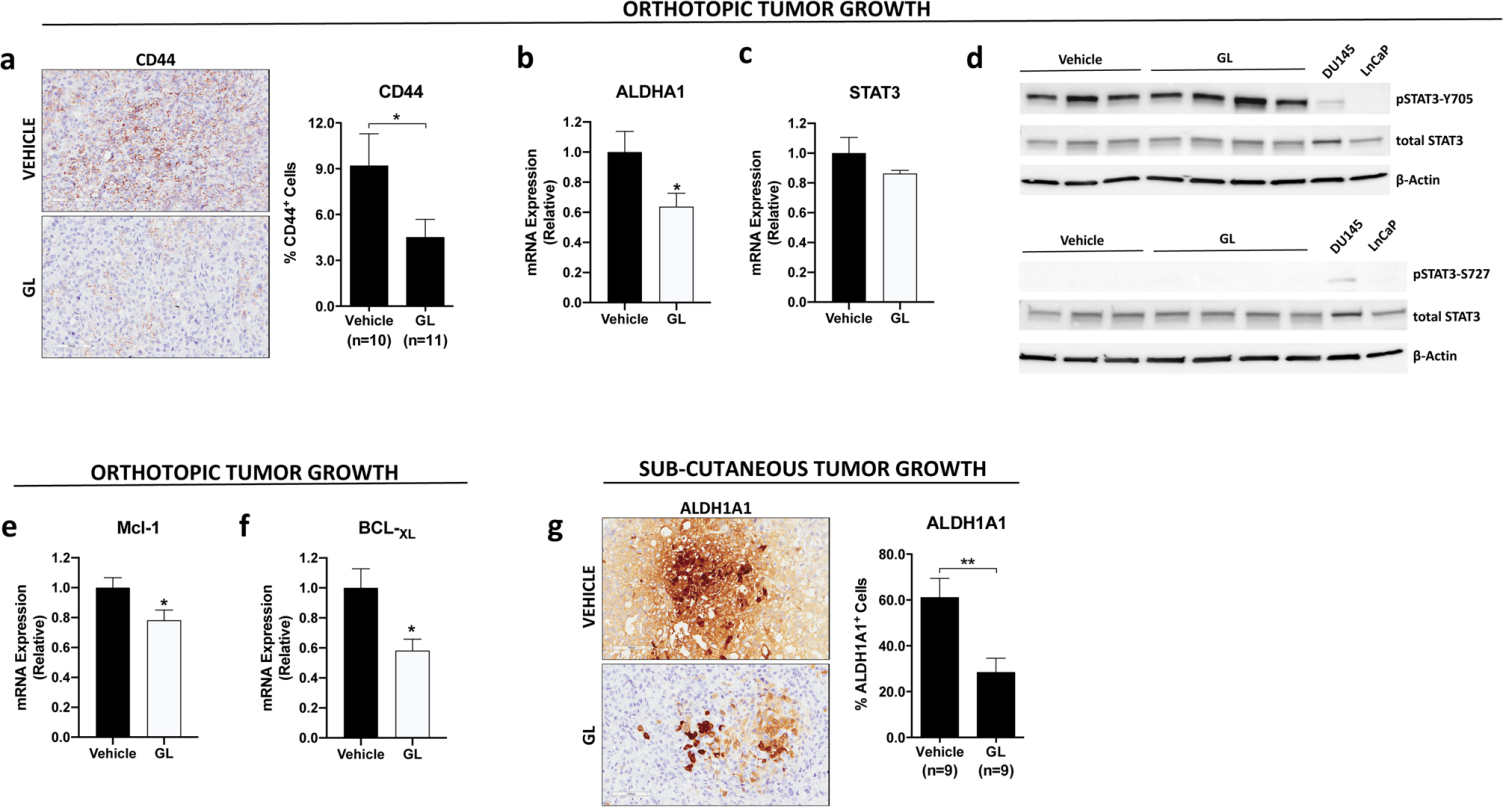
Effect of GL on the expression of STAT3-target genes and on the expression of stemness genes in vivo. (**a**) Representative images and number of CD44^+^ cells in DU145-luc orthotopic xenograft from vehicle- and GL-treated mice. Results are presented as the mean ± s.e.m; statistical significance was determined using an unpaired Student *t* test. *p < 0.05. All images were taken with a 20 T objective (scale bar = 100 mm). (**b**) qPCR analysis of ALDHA1 levels in tumor samples of mice injected orthotopically with DU145-luc cells and treated vehicle- or GL-treated. Results represent the mean ± s.d. of three independent experiments (n = 3), each performed in triplicate. *p < 0.05. (**c**) qPCR analysis of STAT3 levels in tumor samples of mice injected orthotopically with DU145-luc cells and treated with vehicle or GL. Results represent the mean ± s.d. of three independent experiments (n = 3), each performed in triplicate. (**d**) Western blot analysis of pSTAT3-Y705, pSTAT3-S727 and total STAT3 levels in tumor samples of mice injected orthotopically with DU145-luc cells and treated with vehicle or GL. Lysates from DU145 and LnCaP cell lines were used as positive and negative controls, respectively. A representative blot of two independent experiments (n = 2) is shown. (**e**) qPCR analysis of Mcl-1 levels in tumor samples of mice injected orthotopically with DU145-luc cells and treated with vehicle or GL. Results represent the mean ± s.d. of three independent experiments (n = 3), each performed in triplicate. *p < 0.05. (**f**) qPCR analysis of Bcl-_XL_ levels in tumor samples of mice injected orthotopically with DU145-luc cells and treated with vehicle or GL. Results represent the mean ± s.d. of three independent experiments (n = 3), each performed in triplicate. *p < 0.05. (**g**) Representative images and number of ALDH1A1^+^ cells in DU145 sub cutaneous xenografts from vehicle- and GL-treated mice. Results are presented as the mean ± s.e.m; statistical significance was determined using an unpaired Student *t* test. **p < 0.01. All images were taken with a 20 T objective (scale bar = 100 mm).

We next determined the effect of STAT3 inhibition by GL on CSCs and TA/CB cell populations derived from DU145 cells. The treatment with GL significantly compromised the viability of both DU145-CSCs and -TA/CB cells at 24 h and 48 h in a concentration-dependent manner, indicating that both cell populations’ viability depends on STAT3 (Fig. 2a). Since it is known that prostate CSCs are dependent on the JAK-STAT3 signaling pathway for their survival and proliferation^17^, we next sought to determine the potential of GL to disrupt their ability to form colonies and spheres. We performed colony- and sphere-formation assays in the presence of GL on CD133^+^ (CSCs) and CD133^−^ (TA/CB) cells isolated from DU145 cells. We found that GL significantly reduced the number of CSCs- and TA/CB-derived colonies in a concentration-dependent manner: however, CSCs were slightly more sensitive to lower concentrations of GL than TA/CB cells, as their colony-forming ability significantly decreased by almost 50% in the presence of 2.5 µM GL after 24 h (Fig. 2b). The same level of colony-forming inhibition was reached in TA/CB cells only in the presence of 10 µM GL after 24 h or 2.5 and 5 µM GL after 48 h, indicating that this population is slightly more resistant (Fig. 2b). Similarly, GL significant reduction of the number of CSCs-derived spheres was also concentration-dependent (Fig. 2c), while the number of TA/CB-derived spheres was reduced only in the presence of higher concentrations of GL (10 µM) (Fig. 2d). Of note, mRNA expression analysis showed that the STAT3-regulated genes Mcl-1, Bcl-_XL_, c-myc and survivin were significantly down-regulated in CSC-derived spheres treated with GL, supporting the hypothesis that the STAT3 signaling is involved in survival of PCa-CSCs (Fig. 3a). The expression of Mcl-1 and Bcl-_XL_ in TA/CB-derived spheres was significantly decreased only after treatment with 10 µM GL and no effect of GL was observed on c-myc or survivin expression (Fig. 3b), likely reflecting the lower expression of pSTAT3 in TA/CB cells compared to CSCs. We next explored the effects of GL in vivo on the expression of stem cell markers in PCa xenografts in two mouse models that we previously described^22,23^. In the first approach, nude mice were injected orthotopically with DU145-Luc cells and treated with GL or vehicle for 9 weeks^22^. Our IHC analysis of the primary tumors showed that the treatment with GL reduced the expression of the stemness marker CD44 by 40% (Fig. 4a). Furthermore, we found that the expression levels of the stemness marker ALDH1A1 were also decreased in tumors treated with GL compared to vehicle-treated samples (Fig. 4b). The treatment with GL did not affect the mRNA levels of STAT3 (Fig. 4c), nor its protein levels and the phosphorylation of STAT3 on Tyrosine-705 (Y705) and Serine-727 (S727) (Fig. 4d), but it significantly decreased the expression levels of the STAT3-target genes Mcl-1 and Bcl-_XL_ (Fig. 4e-f). These results are in agreement with previous data showing that GL inhibits STAT3 binding to DNA without affecting its phosphorylation status^20^. In the second in vivo approach, DU145 cells were injected subcutaneously and animals subjected to daily intraperitoneal injections of GL or vehicle for 3 weeks^23^. In this model, IHC analysis of the primary tumors showed that the treatment with GL reduced the expression of the stemness marker ALDH1A1 by 50% (Fig. 4g). Interestingly, previous data from our group showed that the treatment with GL in this model also reduced the mRNA expression levels of ALDH1A1 and of the STAT3-target genes Mcl-1 and Bcl-_XL_^21,23^. Taken together, these data indicate that treatment with GL reduces the viability, the colony forming and the sphere-forming efficiency of DU145-CSCs in vitro, and reduces the expression of stemness markers and STAT3-regulated genes in vivo.

### Treatment with GL reduces the viability of docetaxel-resistant and patient-derived spheres with stemness characteristics

**Figure 5:**
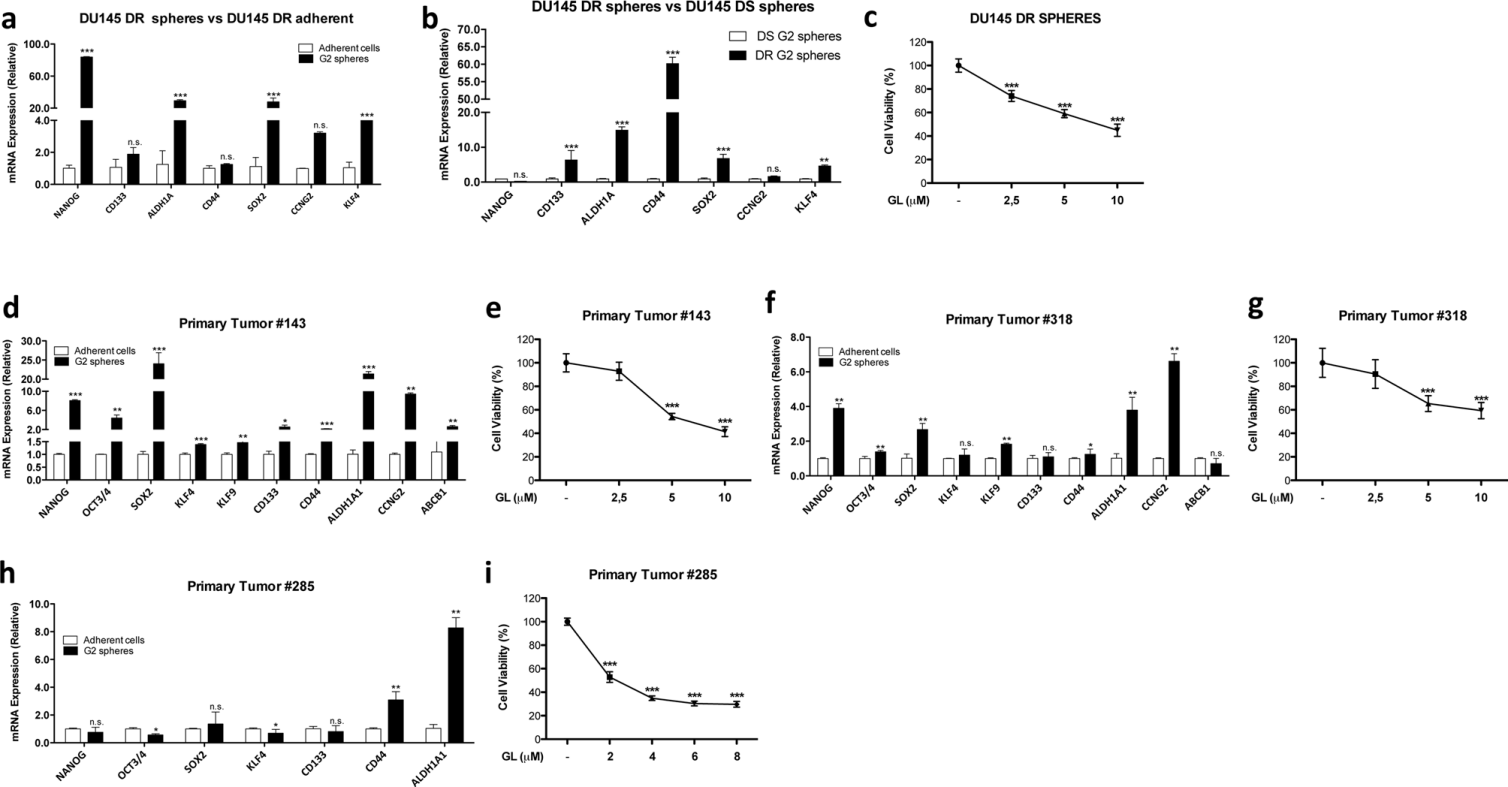
Effect of GL on docetaxel-resistant DU145 spheres and on patient-derived spheres. (**a**) qPCR analysis of stemness related genes in DU145-DR adherent cells and spheres. Results represent the mean ± s.d. of three independent experiments (n = 3), each performed in triplicate. ***p < 0.001; *n.s.* not significant. (**b**) qPCR analysis of stemness related genes in DU145-DR spheres and DU145-DS spheres. Results represent the mean ± s.d. of three independent experiments (n = 3), each performed in sextuplicate. **p < 0.01; ***p < 0.001; *n.s.* not significant. (**c**) Viability assay on spheres derived from DU145-DR cells grown in the presence of vehicle or 2.5–10 μM GL for 48 h. Results represent the mean ± s.d. of six (n = 6) independent experiments, each performed in quintuplicate. Statistical significance was determined using one-way ANOVA with Bonferroni post hoc test. ***p < 0.001. (**d**) qPCR analysis of stemness related gene expressions in spheres and adherent cells derived from primary tumor #143. Results represent the mean ± s.d. of six independent experiments (n = 6), each performed in triplicate. *p < 0.05; **p < 0.01; *n.s.* not significant. (**e**) Viability assay on spheres derived from primary tumor #143 grown in the presence of vehicle or 2.5–10 μM GL. Results represent the mean ± s.d. of seven (n = 7) independent experiments, each performed in quintuplicate. Statistical significance was determined using one-way ANOVA with Bonferroni post hoc test. ***p < 0.001. (**f**) qPCR analysis of stemness related gene expressions in spheres and adherent cells derived from primary tumor #318. Results represent the mean ± s.d. of six independent experiments (n = 6), each performed in triplicate. *p < 0.05; **p < 0.01; *n.s.* not significant. (**g**) Viability assay on spheres derived from primary tumor #318 grown in the presence of vehicle or 2.5–10 μM GL. Results represent the mean ± s.d. of ten (n = 10) independent experiments, each performed in quintuplicate. Statistical significance was determined using one-way ANOVA with Bonferroni post hoc test. ***p < 0.001. (**h**) qPCR analysis of stemness related gene expressions in spheres and adherent cells derived from primary tumor #285. Results represent the mean ± s.d. of six independent experiments (n = 6), each performed in triplicate. *p < 0.05; **p < 0.01; *n.s.* not significant. (**i**) Viability assay on spheres derived from primary tumor #285 grown in the presence of vehicle or 2–8 μM GL. Results represent the mean ± s.d. of six (n = 6) independent experiments, each performed in quintuplicate. Statistical significance was determined using one-way ANOVA with Bonferroni post hoc test. ***p < 0.001.

We next investigated the effect of GL on spheres derived from docetaxel-resistant DU145 cells (DU145-DR) and on spheres derived from three treatment-naïve patients with different Gleason scores, obtained after radical prostatectomy or prostate biopsy at diagnosis (Table 1). As shown in Fig. 5, DU145-DR- and patient-derived spheres were highly enriched in two or more stemness genes compared to the parental cells (Fig. 5a,b,d,f,h), which indicates that these spheres retain stemness characteristics and is consistent with their ability to survive and proliferate in a selective medium without adhering to a solid surface. Interestingly, DU145-DR spheres showed a significant enrichment in stemness genes also compared to spheres derived from docetaxel-sensitive cells (DU145-DS), which corroborates the role of these genes in drug resistance (Fig. 5b). We also detected higher levels of STAT3 expression in both DU145-DS and DU145-DR derived spheres compared to the adherent controls (Suppl. Fig. S1). Of note, the expression of STAT3 decreased in GL treated spheres, which confirms the role of STAT3 in driving sphere formation and stemness features (Suppl. Fig. S1). Both DU145-DR and patients-derived spheres also showed a slower growth rate (Suppl. Fig. S2a-c-e) and a greatly reduced protein synthesis activity (Suppl. Fig. S2b-d-f) compared to the parental cells, in agreement with their CSC phenotype. The viability of DU145-DR spheres was significantly compromised by GL in a concentration-dependent manner, reaching a 50% reduction after treatment with 10 µM GL (Fig. 5c). Spheres derived from patients #143, #318 and #285 responded strongly to GL already at 2–5 µM, showing a significant decrease in viability up to 60%, 40% and 70%, respectively, at 8–10 µM GL (Fig. 5e,g,i). In both DU145-DR and patient-derived spheres, we did not detect any difference between spheres and parental cells in terms of viability after the treatment with GL (data not shown). However, while both DU145-DR spheres and parental cells were sensitive to GL, we found that DU145-DR spheres were more sensitive to GL than DU145-DS spheres (IC_50_ = 6.2 μM and IC_50_ = 10.1 μM, respectively) (Table 2).

**Table 1:** The table summarizes the age, PSA level, Gleason Score and metastasis status of the patients that participated in the study.

Patient information				
Patient #	Age	PSA	Gleason score	Metastasis
143	78	1606	5 + 4	Positive
285	65	6.16	3 + 4	Unknown
318	67	5	4 + 5	Unknown

Patients samples were obtained after radical prostatectomy or prostate biopsy at diagnosis and used to generate prostate spheres for further studies.

**Table 2:** IC_50_ of GL in DU145-DS adherent cells, DU145-DS spheres, DU145-DR adherent cells and DU145-DR spheres. DU145-DR spheres and parental cells were sensitive to GL. However, DU145-DR spheres were more sensitive to GL than DU145-DS spheres [IC_50_ = 6.2 μM and IC_50_ = 10.1 μM, respectively; *p* < 0.05 (*)].

	GL IC_50_ (μM)
DU145-DS adherent cells	8.51
DU145-DS spheres	10.1
DU145-DR adherent cells	6.24
DU145-DR spheres	6.21

Taken together, these data indicate that targeting STAT3 with GL significantly reduces the viability of docetaxel-resistant and patient-derived spheres with stemness characteristics.

## Discussion

In this study we demonstrate that inhibition of activated STAT3 by GL can decrease the viability of docetaxel-resistant and patient-derived spheres retaining stemness characteristics, indicating that this is a promising approach to target the cancer stem cell niche in PCa. The prostate CSCs were identified based on the expression of CD133 and CD44 cell surface markers (CD133^+^/CD44^+^), which allowed us to distinguish CSCs from the more differentiated transient-amplifying (TA) or committed-basal (CB) non-stem cell populations (CD44^+^/CD24^+^)^10–12^. The expression of CD133 and CD44 on the cell surface is considered a marker of stemness in several cancer types, including prostate, as these proteins are involved in cell–cell interactions, cell adhesion and migration of tumor cells^24,25^. Previous studies have reported that CD44^+^ PCa cells from xenografted human tumors are enriched in tumorigenic and metastatic progenitor cells^26^, while CD133^+^ cells have elevated proliferative potential in vitro and the ability to form prostatic-like acini in immunocompromised mice^27^. Additionally, other studies have shown that CD133^+^/CD44^+^ cells display in vitro self-renewal and express core stem cell genes^28–30^. Here we show that CSCs sorted from DU145 cells express high levels of stem cell genes and can form colonies and spheres in vitro: thus, our results are in agreement with previous studies and our model confirms that CD133^+^/CD44^+^ cells retain stemness features. However, our analysis on spheres derived from docetaxel-resistant DU145 cells (DU145-DR) and spheres derived from PCa patients show that the expression of some stem cell markers is increased, while the expression of some others is unchanged. This reflects the high level of heterogeneity often described in the clinic^31,32^. Nevertheless, our spheres express high levels of several pluripotency genes, including *NANOG*, *ALDH1A1*, *SOX2*, *OCT4* and *KLF4*, among others. Several studies have validated the implication of these genes in the regulation of the PCa stem cell niche, which confirms that these spheres retain stemness characteristics^33–37^. Indeed, our data indicate that a pattern of different embryonic pluripotency genes is crucial for the identification of docetaxel-resistant prostate CSCs. Additionally, we show that the PCa CSCs express high levels of active phosphorylated STAT3 (pSTAT3), and that GL down-regulates transcriptional targets of STAT3 in these cells compared to TA/CB non-stem cell populations, indicating STAT3 activity in CSCs. Other groups have demonstrated that the JAK-STAT signaling pathway is constitutively active in the PCa stem-like population and that stem-like and progenitor cells from human PCa patients have constitutively activated STAT3^11,38,39^. Furthermore, gene expression profiling analyses have reported the importance of the JAK-STAT pathway in regulating the stem cell niche in PCa^17^. Thus, in agreement with previously published work, prostate CSCs are dependent on STAT3 signaling, confirming the importance of this pathway for the survival of stem-like cells in PCa.

The current therapeutic strategies for PCa emphasize the elimination of the bulk of the cells within the tumor, which often leads to therapy resistance and activation of normally quiescent CSCs, in turn repopulating the tumor and ultimately promoting disease progression^24,40^. Therefore, developing therapeutics that can selectively target CSCs along with the more differentiated cancer cells is of fundamental importance and represents one of the current challenges in PCa therapy. In this study we demonstrate that inhibition of STAT3 by GL can significantly decrease the viability of CSCs in multiple different models, including docetaxel-resistant and patient-derived spheres. Notably, we found that Docetaxel-resistant spheres were more sensitive to GL than Docetaxel-sensitive spheres (IC_50_ = 6.2 μM and IC_50_ = 10.1 μM, respectively). This could be because the resistant spheres are the result of a selection of cells surviving to the toxic drug treatment (Docetaxel, in this case) and as a consequence these cells are not able to survive to a further challenge with GL. In contrast, the sensitive spheres have not undergone a drug selection treatment and therefore they likely maintain diverse cell subpopulations that may be able to resist to GL. Thus, we provide evidence that GL can target both CSCs and non-CSCs populations, emphasizing the relevance of such an approach for a more effective treatment^19,21^. STAT3 has been demonstrated to contribute to the development of resistance to the anti-androgen enzalutamide and STAT3 inhibition by GL can enhance the effect of anti-androgen therapy as well as decrease proliferation of resistant PCa cells^41^. This study now expands on the potential therapeutic benefits of this small molecule by showing that GL can harness the main bulk of tumor cells as well as the tumor-initiating stem cell niche in which activation of STAT3 is clearly demonstrated. Notably, the fact that DU145-DR spheres are sensitive to GL suggests that GL is a potential drug to be used in combination with a second line therapy for PCa treatment. Moreover, targeting the downstream activity of STAT3 with this approach could be highly beneficial because it would reduce the risks of developing therapy resistance to upstream inhibitors, thus decreasing side effects and increasing efficacy of the therapy.

Taken together, our data confirm that PCa-CSCs are dependent on STAT3 signaling for survival, and that blocking this pathway with GL effectively impairs their viability. Our findings here provide the first evidence that GL can target the CSCs in patient-derived primary prostate tumor cultures. We propose that the STAT3 inhibitor GL acts by reducing the viability of PCa-therapy-resistant cells in both CSCs and non-CSC populations.

## Methods

All experimental procedures and protocols were approved by the Regional Ethical Board at Lund University (Sweden), the Ethic Commitee of Clinic Investigation at the Vall d’Hebron Hospital of Barcelona (Spain) and the Committee of Bioethics of the Faculty of Medicine at the Clinical Hospital of the University of Chile (Chile). All experiments were performed in accordance with relevant guidelines and regulations.

### Cell lines, primary cell lines and reagents

DU145 and LNCaP cells were from the American Type Culture Collection (ATCC, Manassas, VA, USA) and were cultured in RPMI-1640 medium supplemented with 10% fetal bovine serum (FBS) and 1% penicillin/streptomycin. DU145-Luciferase (DU145-Luc) were from Anthem Biosciences (Bangalore, India) and were grown in Dulbecco's Modified Eagle Medium (DMEM) supplemented with 10% fetal bovine serum (FBS) and 1% penicillin/streptomycin. Cells were regularly tested to confirm the absence of *Mycoplasma* infection. The molecular characterization of the cell lines was performed by LGC Standards (Cologne, Germany) and the results were then evaluated by comparison with the ATCC database (https://www.lgcstandards-atcc.org/STR_Database.aspx). Our batches of cells revealed 100% match to the ATCC standard. Docetaxel-resistant DU145 (DU145-DR) cells were developed as previously described^42,43^. DU145-DR cells were cultured in RPMI-1640 (BioWest) supplemented with 10% FBS, 2 mM l-glutamine, 100 U of penicillin/ml, 100 μg/ml of streptomycin and 0.1 mM non-essential amino acids (all from BioWest) in the presence of 2.5 nM of docetaxel (Sigma-Aldrich, St. Louis, MO). Primary cell lines were isolated from human prostate cancer samples. Informed consent‬ was obtained from all patients involved in the study and all methods were carried out in accordance with relevant guidelines and regulation of the local ethics committees that approved the study. The prostate cancer tissue #143 was obtained from a patient biopsy with Gleason Score 9 (5 + 4) at Vall d’Hebron Hospital of Barcelona (Spain), with the approval of the Ethic Commitee of Clinic Investigation n. PR(AG)96/2015. PCa tissues #318 and #285 are derived from radical prostatectomies performed at the Clinical Hospital of the University of Chile with the approval of the Committees of Bioethics of the Faculty of Medicine (N° 075/2013 and N° 48/2013). Tissues were minced into small pieces by mechanical digestion, washed with culture medium and seeded in collagen-I/poly-d-lysin pre-coated 6-well plates. Cells were cultured in DMEM-F12 medium supplemented with 7% heat-inactivated FBS, 2 mM l-glutamine, 100 U of penicillin/ml, 100 μg/ml of streptomycin, 0.1 mM non-essential amino acids, 600 µg/ml glucose, 1 mg/ml transferrin, 250 µg/ml insulin, 100 µg/ml putrescin, 200 ng/ml sodium selenite, 1 mM hydrocortisone, 20 ng/ml EGF, 10 ng/ml βFGF, 200 ng/ml vitamin E and 200 ng/ml vitamin A. All cells were grown in a humidified incubator at 37 °C with 5% CO_2_. Galiellalactone (GL) was provided by Glactone Pharma AB (Helsingborg, Sweden).‬‬‬‬‬‬‬‬‬‬‬‬‬‬

### Cell sorting

For cell sorting, DU145 cells were harvested using a cell dissociation solution (Sigma Aldrich, #C5789) and incubated with antibodies to CD133-PE (#130-090-853), CD44-FITC (#130-095-195) and CD24-APC (#130-095-954), or their corresponding isotype controls (IgG2b-PE #130–092-215, IgG1-FITC #130-092-213, IgG1-APC #130-092-214) for 15 min at 4 °C, according to manufacturer’s instructions. All antibodies were from Miltenyi Biotec. Dead cells were excluded using 7-AAD (BD Pharmingen, #559925). Cell sorting was performed using a BD FACSAria and analyzed with BD FACSDiva software (BD Biosciences). Alternatively, CD133^+^ and CD133^−^ cells were isolated using the CD133 MicroBead Kit (#130-100-857, Miltenyi Biotec). Sorted cancer stem cells (CSCs: CD133^+^/CD44^+^ or CD133^+^) and transient amplifying/committed basal cells (TA/CB: CD44^+^/CD24^+^ or CD133^−^) were collected in RPMI medium and plated for further experiments.

### Immunocytochemistry

Immunocytochemistry for pSTAT3 was conducted on DU145 parental cells and on sorted CSCs and TA/CB cells. Cytospins were obtained after centrifugation at 1,200 rpm during 2 min using 2,000 cells per sample. Expression of pSTAT3 was evaluated using the anti-pSTAT3 antibody (Tyr705, Cell Signaling Technology Inc., Beverly, MA, USA) following standard avidin–biotin immunoperoxidase procedures^22^. Stained slides were scanned using the Aperio ScanScope XT Slide Scanner system (Aperio Technologies, Vista, CA, USA) for bright-field microscopy and 10 × magnification pictures were taken using the same system.

### Clonogenic assay

The clonogenic ability of sorted cells was evaluated as previously described^11,44^. Cells were seeded in 6-well collagen-coated plates (Gibco, Life Technologies) at a density of 0.8 × 10^3^ cells/well in the presence of vehicle or increasing concentrations of GL (0–10 µM). After 24 h or 48 h medium was changed to complete medium and cells were let growing until colonies were spotted in the vehicle-treated group. Colonies were scored using the ImageJ software if they contained more than 50 cells (6 population doublings), usually between 10 and 20 days after treatment.

### In vitro sphere formation assay

For sphere formation assays showed in Figs. 1d and 2c,d, 1 × 10^5^ cells were grown in Prime-XV Tumorsphere SFM medium (Irvine Scientific) supplemented with 2 U/ml Heparin (Sigma Aldrich, #H3149) and 0.5 μg/ml hydrocortisone (Sigma Aldrich, #H0135). Where indicated, cells were grown in the presence of vehicle or increasing concentrations of GL (0–10 µM). Cells were grown in Ultra-Low attachment multiwell plates (Corning) for 5–10 days, until spheres were clearly visible. Spheres in each well were then counted manually under the microscope and 10 × magnification pictures were taken using the same system.

For data presented in Fig. 5, spheres were generated from docetaxel-sensitive and docetaxel-resistant DU145 cells and from primary cell lines by seeding single cells (2 × 10^6^/dish) in 15 cm dishes pre-treated with Poly-2-hydroxyethyl methacrylate (PolyHEMA) (Sc Quimigen), in order to inhibit cell attachment. Cells were plated in serum-free DMEM-F12 medium containing 2 mM l-glutamine, 100 U of penicillin/ml, 100 μg/ml of streptomycin, 0.1 mM non-essential amino acids, 600 µg/ml glucose, 1 mg/ml transferrin, 250 µg/ml insulin, 100 µg/ml putrescin, 200 ng/ml sodium selenite, 1 mM hydrocortisone, 20 ng/ml EGF, 10 ng/ml βFGF, 200 ng/ml vitamin E, 200 ng/ml vitamin A and 0.4% B27 (Invitrogen). Spheres were allowed to grow for 3 days (G1), then dissociated and re-plated in the same conditions for 3 more days (G2).

### Growth curves and cytotoxic assays

Cell viability in Fig. 2a was measured using the WST-1 assay, as previously described^45^. Briefly, 2 × 10^3^ CSCs and TA/CB cells were plated in 96 well plates in triplicate and cells were allowed to adhere for 24 h. The cells were then incubated with the vehicle or with increasing concentrations of GL (0–10 µM) for 24 h and 48 h. WST-1 reagent (Roche Applied Science) was added to each well according to the manufacturer’s instructions and the absorbance was measured at a wavelength of 450 nm and 630 nm as a reference using ELISA plate reader.

Cell growth and cell viability in Fig. 5 and Suppl. Fig. S2 were measured by using the crystal violet assay. For growth curves showed in Suppl. Fig. S2, cells (1.5 × 10^3^/well) were seeded on collagen-coated 96-well plates in octuplicates. For cytotoxic assays showed in Fig. 5, 5 × 10^3^ DU145-DR cells/well or 2 × 10^3^ primary cultures/well were seeded on collagen-coated 96-well plates in quintuplicate. After 24 h, cells were treated with vehicle (PBS) or with increasing concentrations of GL (0–10 µM) for 48 h. At the indicated time points cells were fixed in 4% formaldehyde solution, washed with PBS and stained with 0.5% crystal violet for 20 min. Crystals were then dissolved with 15% acetic acid and optical density was read at 595 nm.

### RNA extraction, reverse transcriptase PCR and real-time PCR

RNA extraction, reverse transcriptase PCR and quantitative real-time PCR (qPCR) were performed as previously described^46^. Briefly, total RNA from cells or tumors was extracted using Trizol (Life technologies) and the RNeasy kit (Qiagen) according to the manufacturer’s instructions. One to two micrograms of RNA were used for cDNA synthesis using random primers and the RevertAid RT kit (Thermo Scientific) or the Mooney Murine Leukemia Virus Reverse Transcription (M-MLV-RT) kit (Promega, Madison, USA). Real-time qPCR analysis was performed on a Stratagene Mx3005P system (Agilent Technologies) using Maxima SYBR Green/Rox according to the manufacturer’s instructions (Thermo Scientific), or with the Universal Probe Library (Roche, Basel, Switzerland) on a LightCycler 480 RealTime PCR instrument (Roche). The ΔΔCt method was applied to estimate relative transcript levels. The relative expression of studied genes was normalized to the expression of the β-actin, TBP or IPO8 genes. The primers used are listed in Table 3. Values are presented as mean + SD.

**Table 3:** The table reports the sequences (FW and RV sequence) of the primers used for quantitative real-time PCR (qPCR).

Gene name	Primer FW (5′–3′)	Primer RV (5′–3′)
STAT3	GCTTCCTGCAAGAGTCGAATG	TGTAGAAGGCGTGATTCTTCCC
CD133	TGGGGCTGCTGTTTATTATTCT	TGCCACAAAACCATAGAAGATG
CD44	AGAAGGTGTGGGCAGAAGAA	AAATGCACCATTTCCTGAGA
CD24	CTCCTACCCACGCAGATTTATTC	TGGTGGCATTAGTTGGATTTGG
ALDHA1	TCCTGGTTATGGGCCTACAG	CTGGCCCTGGTGGTAGAATA
c-myc	TCAAGAGGCGAACACACAAC	GGCCTTTTCATTGTTTTCCA
Mcl-1	AAGGATGGGTTTGTGGAGTTCTT	GCAAAAGCCAGCAGCACAT
Bcl-XL	TCCTTGTCTACGCTTTCCACG	GGTCGCATTGTGGCCTTT
Survivin	ACCTGAAAGCTTCCTCGACA	TAACCTGCCAATGGAACCTC
β-actin	GCTCGTCGTCGACAACGGCTC	CAAACATGATCTGGGTCATCTTCTC
ABCB1	TGTGGGAAGAAGAGCACAGTGG	TTTATTTCTTTGCCATCAAGCA
ALDH1A1	GCTCTCCACGTGGCATCT	GCCCCATAACCAGGAACAAT
CCNG2	GGGGGTTGTTTTGATGAAAGT	TTGATCACTGGGAGGAGAGC
CD44	CAAGCAGGAAGAAGGATGGAT	AACCTGTGTTTGGATTTGCAG
CD133	GGAAACTAAGAAGTATGGGAGAACA	CGATGCCACTTTCTCACTGAT
KLF4	GCCGCTCCATTACCAAGA	TCTTCCCCTCTTTGGCTTG
KLF9	CTCCGAAAAGAGGCACAAG	CGGGAGAACTTTTTAAGGCAGT
IPO8	CAAATGTGGCAGCTTCTAGGT	ATGCAGGAGAGGCATCATGT
NANOG	ATGCCTCACACGGAGACTGT	AGGGCTGTCCTGAATAAGCA
OCT3/4	GTGCCTGCCCTTCTAGGAAT	GGCACAAACTCCAGGTTTTCT
SOX2	ATGGGGTTCGGTGGTCAAGT	GGAGGAAGAGGTAACACAGG
TBP	GAACATCATGGATCAGAACAACA	ATAGGGATTCCGGGAGTCAT

### Western blotting

Protein extractions, determinations of protein concentration and western blots were performed as previously described^47^. Briefly, 40 μg of total proteins were prepared in 4 × Laemli buffer and heated to 95 °C for 5 min prior to loading on an SDS-PAGE gel. After separation and transfer of the proteins to PVDF membranes, the membranes were blocked and probed with the following antibodies: rabbit anti-pSTAT3-SER727 (#9134, Cell Signaling Technology, 1:1,000), rabbit anti-pSTAT3-Y705 (#76315, Abcam, 1:5,000), rabbit anti-tSTAT3 (#4904S, Cell Signaling Technology, 1:1,000), mouse anti-ß-Actin (clone AC-15, #A5441, Sigma-Aldrich, 1:5,000). After washing, the membranes were incubated with goat anti-rabbit/mouse HRP-conjugated secondary antibodies (Dako). Following a second wash, the separated protein bands were visualized using the Immobilon Western Chemiluminescence HRP substrate (Millipore) and were imaged and analyzed using the ChemiDoc imaging system (Bio-Rad).

### Surface sensing of translation (SUnSET)

The global protein synthesis ability of parental cells and G2 spheres was evaluated by puromycin immunodetection using the SUnSET assay^48^. Cells (3 × 10^5^) were seeded in 6 well plates and 24 h later were treated with 10 μg/ml puromycin for 10 min. Non-treated cells were used as negative control; cells treated with 10 μg/ml puromycin and 10 μg/ml cycloheximide for 10 min were used as positive control. Cell lysates were collected and analyzed by Western blotting as previously described^42^. Antibodies used were goat anti-actin (I-19) (Santa Cruz Biotechnology) and Mouse Puromycin (12D10) (Millipore).

### Immunohistochemistry on prostate tumor xenografts

Immunohistochemistry (IHC) analysis was performed on paraffin embedded DU145 sub cutaneous and DU145-luc orthotopic xenografts from previous studies^22,23^. In the DU145-luc orthotopic in vivo experiment the mice were treated intraperitoneally with vehicle (2% DMSO in PBS) or 5 mg/kg GL (n = 10) for 9 weeks^22^; in the DU145 sub cutaneous xenograft model the mice were treated with vehicle (1% EtOH in PBS) or 3 mg/kg GL (n = 9) for 3 weeks^23^. Animals were euthanized at the end of treatment and primary tumors were dissected, fixed in 10% formalin and embedded in paraffin for immunohistochemical staining. The Malmö-Lund Ethical Committee approved all mouse experiments and experimental procedures (permit n. M29-13 and M148-04). All experiments were performed in accordance with relevant guidelines and regulations.

For IHC analysis, tissue sections from the primary tumors were processed and analyzed to evaluate the expression of stemness-related genes. IHC staining was conducted as previously described^47^ using the EnVision Flex kit and Autostainer Plus (Dako, Glostrup, Denmark), according to the manufacturer’s instructions. The following antibodies were used: anti-human CD44 (156-3C11, Thermo Scientific, CA, USA), anti-human ALDH1A1 (HPA002123, Sigma Aldrich). For each stained slide, 20 × magnification pictures were taken using the Aperio ScanScope XT Slide Scanner (Aperio Technologies, Vista, CA, USA) system for bright field microscopy. The number of positive cells per section was calculated using the Image Scope software (Aperio). CD44 membrane staining was quantified using the membrane v1.1 algorithm within the Halo image analysis software (Indica Labs, Corrales, NM, USA).

### Statistical analysis

All statistical analyses were performed using GraphPad Prism software (GraphPad Software Inc., La Jolla, CA, USA), and statistical significance was determined using analysis of variance (ANOVA) or Student *t* test. More details regarding the specific statistical analyses that were used for each experiment are reported in the figure legends.

## Supplementary information

Supplementary Information.
