# Supplementary material for: STAT3 inhibition with galiellalactone effectively targets the prostate cancer stem-like cell population
Source: Sci Rep. 2020 Aug 18;10:13958. doi: 10.1038/s41598-020-70948-5 (PMC7434889; doi:10.1038/s41598-020-70948-5)
Supplement: Supplementary file 1 — Supplementary Information. [file 41598_2020_70948_MOESM1_ESM.pdf]

Supplementary Information

STAT3 inhibition with Galiellalactone effectively targets the prostate cancer stem-like cell population.

Giacomo Canesin, Valentina Maggio, Macarena Palominos, Anna Stiehm, Hector R. Contreras, Enrique A. Castellón, Juan Morote, Rosanna Paciucci, Norman J. Maitland, Anders Bjartell and Rebecka Hellsten.

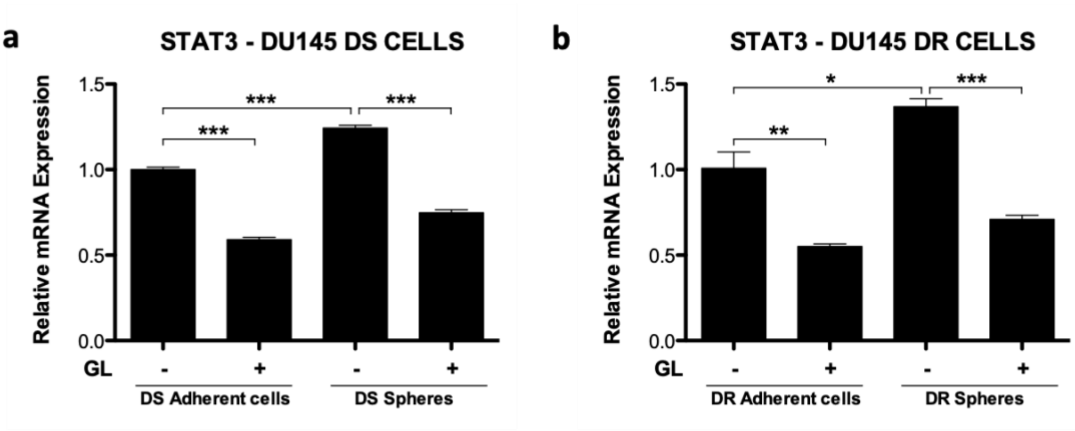

Suppl. Fig. S1. Expression of STAT3 in DU145-DS and DU145-DR spheres and adherent cells.

(a-b) Relative mRNA levels of STAT3 in DU145-DS **(a)** and DU145-DR **(b)** adherent cells or spheres untreated or treated for 24 hours with the respective GL IC_{50}. TBP and IPO8 were used as housekeeping genes. Results represent the mean ± s.d. of two experiments (n=2), each performed in sextuplicate. *p < 0.05, **p < 0.01, ***p < 0.001.

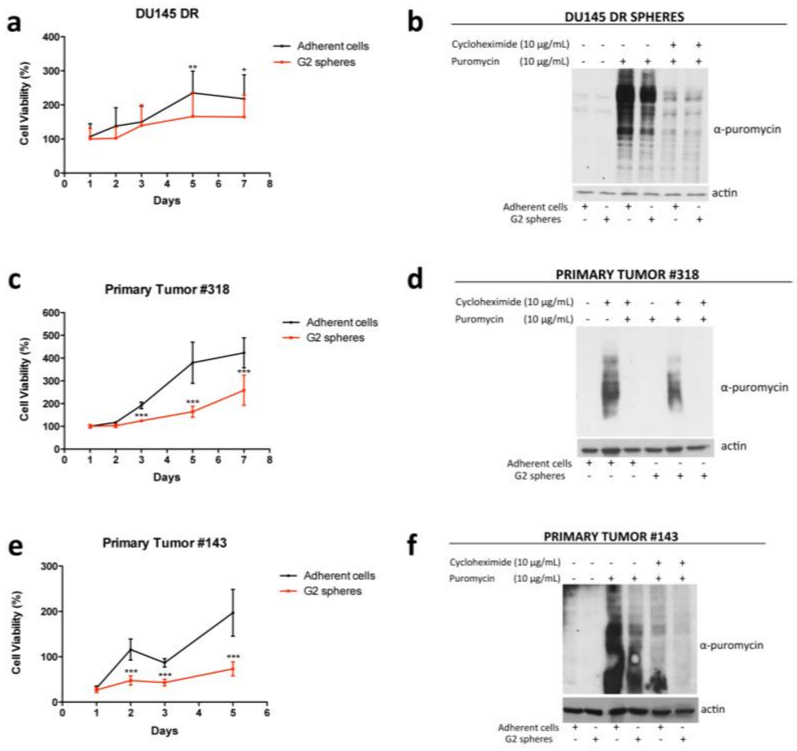

Suppl. Fig. S2. Characterization of docetaxel-resistant spheres and patients-derived spheres.

(a-c-e) Growth curve of DU145-DR adherent cells and spheres **(a)**, spheres and adherent cells derived from primary tumor #318 **(c)** and from primary tumor #143 **(e)**. Results represent the mean ± s.d. of eight (n = 8) independent experiments. Statistical significance was determined using two-way ANOVA. *p < 0.05; **p < 0.01; ***p < 0.001. **(b-d-f)** Evaluation of the global protein synthesis ability by puromycin SUnSET assay in DU145-DR adherent cells and spheres **(b)**, spheres and adherent cells derived from primary tumor #318 **(d)** and from primary tumor #143 **(f)**.

a

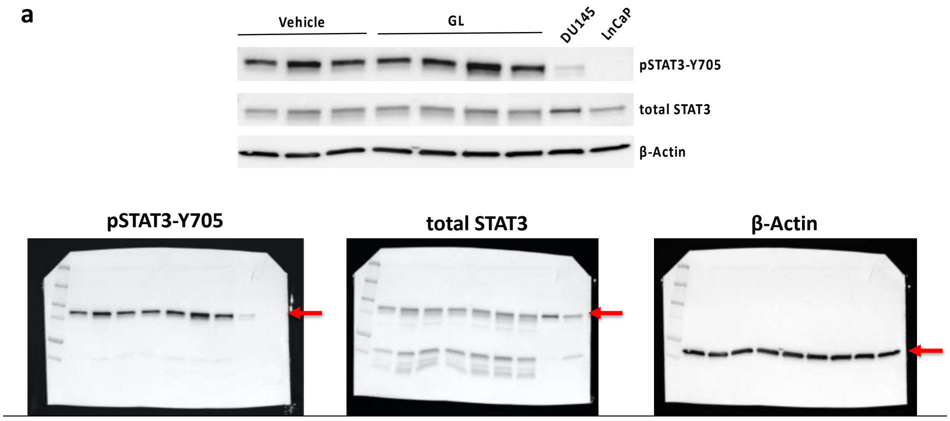

b

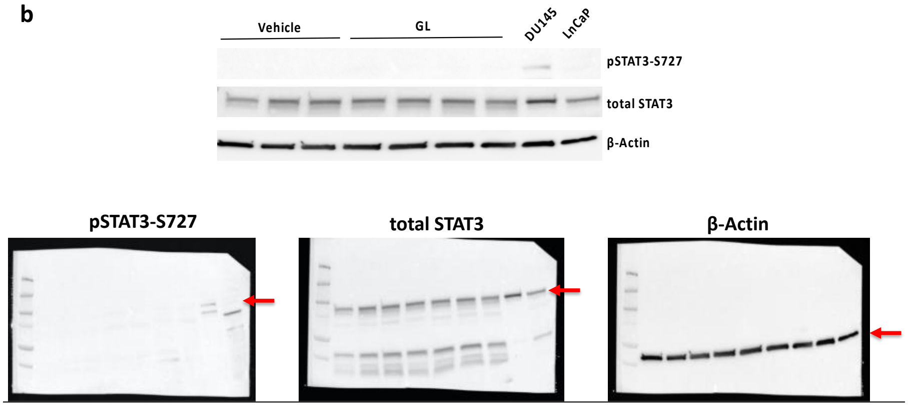

Suppl. Fig. S3: Original blots for data shown in Fig.4d.

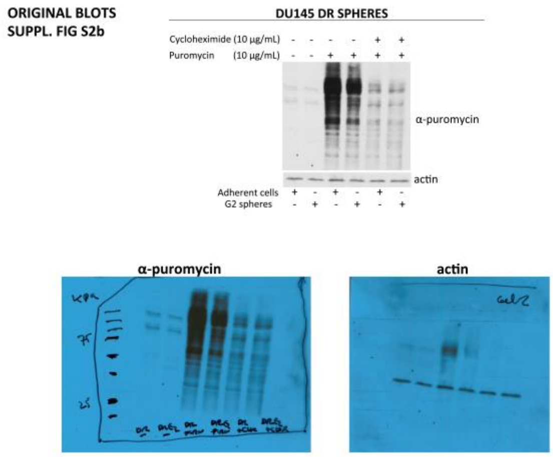

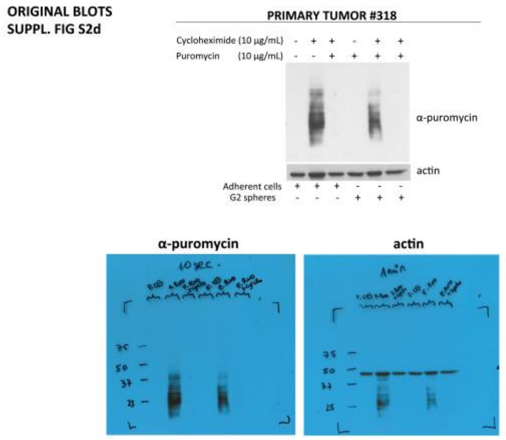

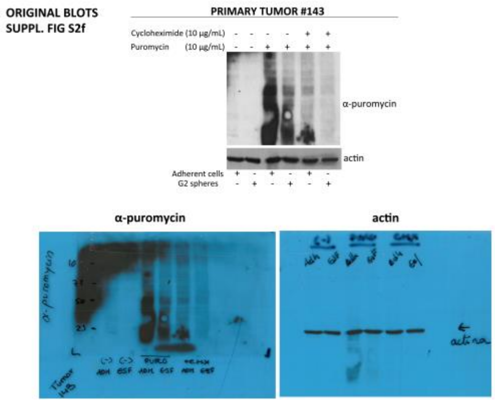

Suppl. Fig. S4: Original blots for data shown in Suppl. Fig.S2.
